# Thermal Insulating and Mechanically Strong Polyimide Aerogel Composites Reinforced by Polyhedral Oligomeric Silsesquioxane-Grafted Carbon Nanotubes

**DOI:** 10.3390/polym17030332

**Published:** 2025-01-25

**Authors:** Yating Wang, Ruirui Yang, Zhe Zhang, Zicheng Shan, Liying Zhang

**Affiliations:** 1 Luoyang Ship Material Research Institute, 169 South Binhe Road, Luoyang 471023, China; wangyt1232023@163.com (Y.W.); cute725@126.com (R.Y.); 2 Shanghai Collaborative Innovation Center of High Performance Fibers and Composites (Province-Ministry Joint), Center for Civil Aviation Composites, Donghua University, Shanghai 201620, China; 2230748@mail.dhu.edu.cn (Z.Z.); 2240562@mail.dhu.edu.cn (Z.S.)

**Keywords:** polyimide aerogels, multiscale pore structure, thermal insulation, mechanical property

## Abstract

In the ship industry, developing thermal insulation materials with exceptional high-temperature resistance, structural stability and light weight is essential. Herein, polyimide (PI) composite aerogels were synthesized. Carbon nanotubes (CNTs) introduced cross-linking structures within the aerogel matrix, effectively reducing shrinkage and forming micrometer-scale pores. Furthermore, the rigid cage-like structure of polyhedral oligomeric silsesquioxane (POSS) generated additional nanoscale pores. This multiscale pore structure enhanced both compressive strength and thermal insulation properties. The PI-CNT-POSS composite aerogel with a 2 wt% CNT content (PI-CP2) demonstrated outstanding overall performance, with compressive strength, modulus and thermal conductivity values of 167.7 KPa, 360.3 Kpa and 40.6 mW/(m·K), respectively, possessing remarkable advantages over the neat PI aerogel. Consequently, this PI composite aerogel can be used as a promising material for heat management in complex environments.

## 1. Introduction

Polyimide (PI) has attracted a lot of attention due to its excellent high-temperature resistance properties [1,2], especially in the fields of shipping, automotive manufacturing and electronic devices [3]. Because PI aerogels combine the nature of a PI network with a high porous structure, they not only exhibit good thermal insulation properties [4] but also maintain structural stability at extreme temperatures [5]. However, PI aerogels undergo significant shrinkage during gelation and drying processes, which is detrimental to their mechanical properties [6,7]. To solve this problem, several approaches have been employed. Wu et al. [8] incorporated silica (SiO_2_) into the PI backbone during gelation to greatly reduce ~16.2% of the shrinkage in the resulting PI aerogels. Li et al. [9] inhibited ~6.5% of shrinkage by adding NH_2_-SiO_2_@Fe_3_O_4_ into the PI aerogels. Zhu et al. [10] prepared a lightweight and robust PI aerogel by cross-linking surface-modified carbon nanotubes (CNTs) with PI chains, achieving the reduction of ~11.3% of the shrinkage in the aerogels. Wu et al. [11] introduced graphene oxide (GO) and nanoscale zirconia (ZrO) as nanofillers into PI aerogels. As a result, the shrinkage in the aerogel decreased by ~10.3%. Inhibiting shrinkage facilitates the maintenance of pore structures and thus reduces the thermal conductivity of aerogels.

In particular, porosity is a crucial factor influencing the thermal insulation properties of aerogels [12]. Higher porosity makes gaseous conduction the dominant mechanism, reducing solid-phase conduction and thereby reducing overall thermal conductivity. Therefore, the construction of PI aerogels with high porosity is essential for high thermal insulation performance. Sun et al. [13] prepared Ti_3_C_2_T_x_ MXene/PI nanofiber (PINF) aerogels with multi-level pore structures. Values of 98.8% for porosity and a corresponding 35.2 mW/(m·K) for thermal conductivity were achieved. Xue et al. [14] used polyvinyl alcohol (PVA) as pore modifier to prepare lightweight, ultra-insulating PI aerogel fibers, which had a porosity of 95.6% and a thermal conductivity of only 28.7 mW/(m·K). Polyhedral oligomeric silsesquioxane (POSS) is a nano-sized material that consists of Si-O-Si linkages, forming nanoporous cage structures [15]. Because of its porous structures, it has been considered a favorable nano-reinforcement for thermal insulting materials [16]. Wu et al. [17] used POSS and cyclic trapezoidal poly (aminophenyl) sesquioxane (PAPSQ) as cross-linkers to prepare PI aerogels with a porosity of 90.0–91.0% and a thermal conductivity of about 29.2 mW/(m·K). Yang et al. [18] introduced PAPSQ as a cross-linker into PI aerogel to prepare an ultra-light and low-thermal-conductivity PI aerogel. Through adjustments to the content of PAPSQ, the thermal conductivity of PI-PAPSQ was as low as 22.9 mW/(m·K), which was about 2.2 mW/(m·K) lower than that of ordinary PI aerogels. However, POSS was very difficult to disperse in the polymer system owing to its large specific surface area and poor compatibility with polymers. Moreover, incorporating raw POSS material could not solve the shrinkage problem of aerogels [19].

In this work, we used CNTs grafted with POSS (CP) as a nano-reinforcement to prepare a PI aerogel with low shrinkage and high porosity. The thermal and mechanical properties of PI composite aerogels were investigated. The effects of CNT and CP contents on the microstructure and the relationship between the structure and properties of the PI composite aerogels were explored. Within the PI composite aerogel framework, CNTs played a reinforcing role in strengthening the skeleton, resulting in better shrinkage resistance. Additionally, the porosity of PI aerogels was improved by grafting porous POSS onto the CNT surface. The increase in porosity played a crucial role in increasing the solid–gas interface, thereby improving thermal insulation performance.

## 2. Materials and Methods

### 2.1. Materials

4,4′-diaminodiphenyl ether (ODA), 1,2,4,5-Benzenetetracarboxylic anhydride (PMDA, 98%), N,N-dimethylformamide (DMF), N, N-dimethylacetamide (DMAc), triethylamine (TEA), tetrahydrofuran (THF) and methanol were supplied by Sinopharm Chemical Reagent Co., Ltd. (Shanghai, China). Multi-walled CNTs were supplied by Nanjing XFNANO Materials Tech Co., Ltd. (Nanjing, China). AminopropylIsobutyl POSS was supplied by Hybrid Plastics, Inc. (Hattiesburg, MS, USA). Deionized (DI) water was used throughout the experiments.

### 2.2. Preparation of POSS-Functionalized CNTs

The raw CNTs were first modified to introduce functional groups on their surface. The CNTs were acidified by impregnating them with the mixture of H_2_SO_4_ (300 mL) and HNO_3_ (100 mL) at 70 °C for 3 h. The acidified CNT (a-CNT) was washed with deionized water repeatedly until the pH stabilized at neutral. Finally, a-CNTs were obtained by vacuum filtration, followed by drying at 60 °C for 48 h in an oven. Afterward, 0.2 mg a-CNT was dispersed in 20 mL of THF and sonicated at 40 kHz for 0.5 h to ensure uniform dispersion. THF was the selected solvent, with a certain polarity that promoted the interaction of the surface functional groups of a-CNT with the solvent, thus improving its dispersion. Subsequently, 0.6 g POSS was slowly added to the mixture. The reaction was carried out under a N_2_ atmosphere at 80 °C for 24 h. The product was collected by washing several times with methanol. The POSS-functionalized CNT (CP) was finally obtained after oven-drying at 60 °C for 48 h.

### 2.3. Preparation of Polyamide Acid (PAA)

A total of 102 g of DMAc was used to dissolve 8.62 g of ODA. Then, 9.40 g of PMDA was gradually added, and the mixture was stirred for 8 h. Subsequently, 4.40 g of TEA was introduced, and stirring continued for 5 h, yielding the PAA solution.

### 2.4. Preparation of PI Composite Aerogels

Certain amounts of CP were sonicated in 25 mL of DI water at 40 kHz for 0.5 h to achieve dispersion. Afterward, the mixture of PAA (1.2 g), CP suspension and TEA (0.6 mL) was stirred for 2 h. The resulting mixture was then moved to a mold which was placed in liquid nitrogen for freezing. The specimens were placed in a freeze dryer at −40 °C for 72 h, ensuring that all solvents were removed and forming a highly porous aerogel structure. Finally, the composite aerogels were obtained by thermal imidization in a tube furnace at 100, 200 and 250 °C for 1 h under a nitrogen atmosphere. PI aerogels with different CP contents were named PI-CP1 (1 wt%) and PI-CP2 (2 wt%). Similarly, PI-C1 and PI-C2 refer to the PI aerogels with 1 and 2 wt% CNT, respectively.

### 2.5. Characterization

FTIR (BRUKER-TENSOR 27, Bruker (Beijing) Scientific Technology Co., Ltd., Beijing, China) was used to characterize the chemical structure. The spectra were collected in the wavenumber range of 4000 to 500 cm^−1^ to identify functional groups and confirm the successful grafting of POSS onto the CNTs. The crystal structure was analyzed by X-ray diffraction (XRD, D/max2550VB3+/PC, Rigaku, Tokyo, Japan). The XRD patterns were recorded from 5° to 60° to ensure the successful grafting of POSS. Thermogravimetric analysis (TGA, PerkinElmer TGA 4000, PerkinElmer, Waltham, MA, USA) was used to evaluate the thermal stability and composition of the composite aerogels. Samples were heated in an air atmosphere from room temperature to 800 °C at a rate of 10 °C/min with a gas flow of 20 mL/min. Surface morphology was observed using field-emission scanning electron microscopy (FESEM, Hitachi S-4800, Hitachi, Tokyo, Japan) at 5 kV. To facilitate observation and ensure optimal imaging quality, the aerogel samples were cut into appropriate sizes and pasted onto a cross-sectional sample stage. Subsequently, the samples were coated with a thin layer of gold using ion beam sputtering (IBS, Cressington 208R, Ted Pella, Inc., Redding, CA, USA) to enhance their electrical conductivity, enabling high-resolution imaging. The volume shrinkage of the aerogels was calculated according to the equation (V_1_ − V_0_)/V_1_ × 100%, where V_1_ and V_0_ are the volumes of the aerogel before and after processing, respectively. The porosity of the aerogels was calculated by the equation (1 − ρ/ρ_sk_) × 100%, where ρ and ρ_sk_ are the densities of the bulk and the skeleton of the aerogels, respectively. The compression mechanical properties were evaluated with a mechanical tester (SANS CMT4104, MTS., Shanghai, China). The thermal conductivity was determined using a thermal analyzer (Hot Disk TPS 250S, Hot Disk, Shanghai, China), while the temperature of the aerogels was monitored with a thermal infrared camera (FLIR, T1050sc, Teledyne FLIR, Thousand Oaks, CA, USA).

## 3. Results and Discussion

**Figure 1:**
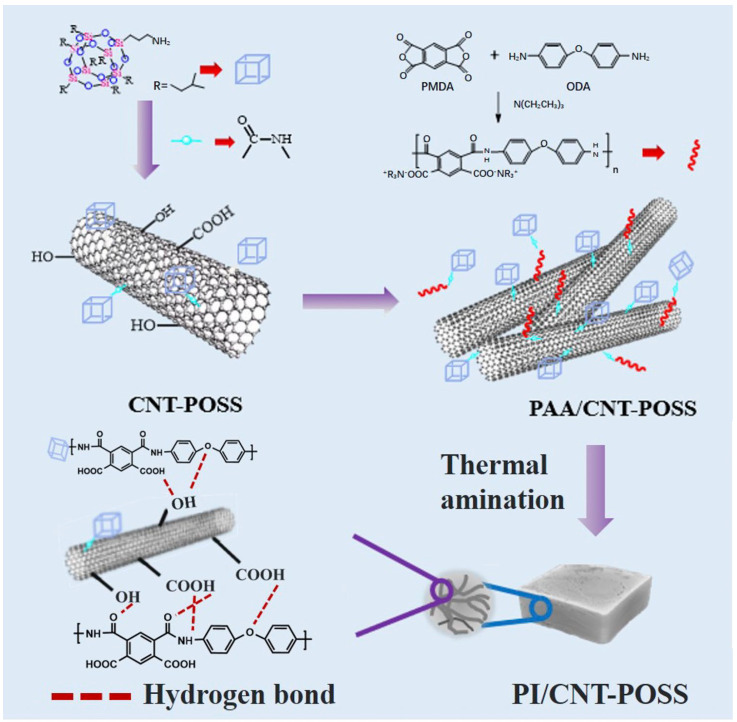
Schematic illustration of the preparation process for PI-CP composite aerogels.

The synthesis route of the PI-CP composite aerogel is shown in Figure 1. First, carboxyl and hydroxyl groups were grafted on CNTs by acidification. POSS was then grafted onto a-CNTs to obtain CP through the reaction of an amino group with a carboxyl group. Meanwhile, PAA precursor was prepared by a polycondensation reaction between PMDA and ODA. Finally, PI-CP composite aerogels were obtained by mixing CP with the PAA precursor solution, followed by freeze-drying and thermal imidization. Within the framework of the composite aerogels, the hydrogen bonding between the functional groups on CNT and PI molecular chains formed a cross-linked structure, which could effectively overcome swelling and capillary forces, thereby inhibiting the shrinkage during gelation and thermal imidization.

### 3.1. Composition and Structure of PI Composite Aerogels

**Figure 2:**
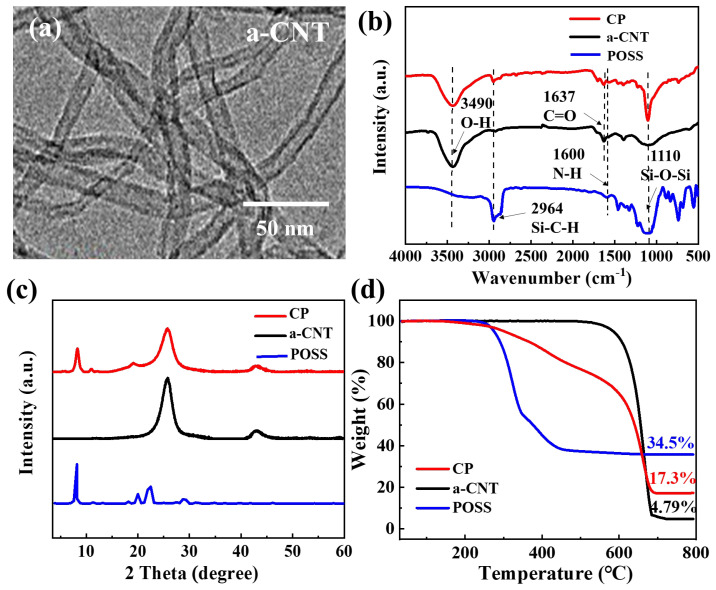
(**a**) TEM image of a-CNT. (**b**) FT-IR spectra of POSS, a-CNT and CP; (**c**) XRD pattern of POSS, a-CNT and CP; and (**d**) TG curve of POSS, a-CNT and CP.

Figure 2a shows the nanotube-like structure of an a-CNT with a diameter of approximately 10 nm. The high aspect ratio and functional groups on a-CNTs can effectively enhance their interaction with the aerogel matrix, thereby affecting the final structure and properties of the aerogel [20]. Figure 2b shows the FTIR spectra of POSS, a-CNT and CP. The peaks located at 2964 and 1110 cm^−1^ were assigned to the stretching vibrations of Si-C-H and Si-O-Si on POSS, respectively [21]. The peaks at 3490 and 1637 cm^−1^ corresponded to the stretching vibrations of the O-H and C=O functional groups of a-CNTs, respectively [22]. The observation of the CP spectrum revealed that the characteristic groups of both POSS and a-CNT were present, suggesting that POSS was successfully grafted onto the a-CNT. Figure 2c displays the crystal structures of POSS, a-CNT and CP. The diffraction peaks of POSS at 9.8°, 21.2° and 23.6° corresponded to the crystal planes of (101), (020) and (110), respectively [23]. The peaks at 25.5° and 43.0° originated from a-CNTs assigned to the crystal planes of (002) and (101), respectively [24]. Through observation of the XRD spectra of CP, the characteristic diffraction peaks of both a-CNTs and POSS appeared, further indicating that POSS was successfully grafted onto a-CNTs. The TGA curves are shown in Figure 2d, where significant weight loss for POSS occurred in a step between 280 °C and 480 °C. The weight loss between 280 and 340 °C corresponded to the thermal degradation of the -NH_2_ groups, whereas the weight loss between 340 and 480 °C occurred as a result of the thermal decomposition of isobutyl moieties of POSS [25]. The solid residue was around 34.5% due to the conversion of siloxane to silica. The a-CNT samples experienced weight loss at ~600 °C. The weight loss was 95.2% due to the escape of CO_2_ produced by the reaction of C with O_2_ in the air. The residual weight percentage of 4.8% was attributed to the magnetic particles remaining in the CNT during preparation [26]. For the TGA curve of the CP sample, the decomposition of POSS occurred from 280 to 480 °C and the escape of CO_2_ happened from 480 to 680 °C [27], resulting in a final residual weight of 17.3%. According to the above data, the weight percentage of POSS in CP was 17.3%/95.2% = 18.2%.

**Figure 3:**
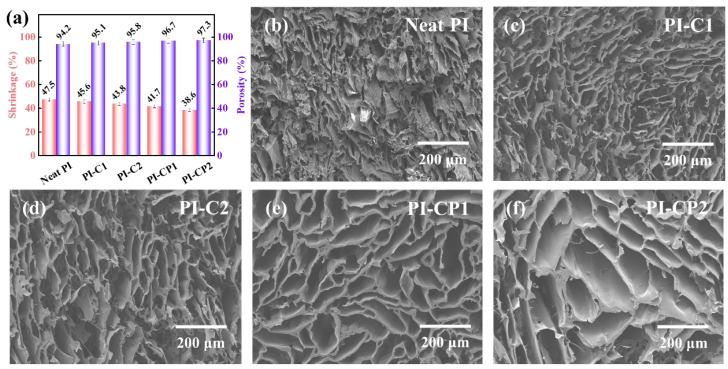
(**a**) Volume shrinkage and porosity of PI aerogels; SEM images of (**b**) neat PI, (**c**) PI-C1, (**d**) PI-C2, (**e**) PI-CP1 and (**f**) PI-CP2.

Shrinkage and porosity are particularly important for the structural stability and thermal insulation of aerogels [28]. However, the drying and thermal imidization of PI aerogels causes severe shrinkage and seriously affects the pore structure of the materials [29,30]. Table 1 summarizes the density, volume shrinkage and porosity of all the samples. These data reflect the physical properties of the PI aerogels and the influence of the components on the aerogel structure. The variation trends in shrinkage and porosity for different aerogels are shown in Figure 3a. Through the addition of CNTs into the PI matrix, the shrinkage of aerogel was reduced from 47.5% (neat PI) to 45.6% (PI-C1). The shrinkage of the aerogel further decreased to 43.8% (PI-C2) as the content of CNT increased. This change can be attributed to the interaction between functionalized CNT and PI chains. Furthermore, CNTs acted as a bridge, alleviating the capillary force and thermal stress shock during ice sublimation which helped minimize the shrinkage of the aerogels. In addition, compared with PI-C1, the shrinkage of PI-CP1 decreased to 41.7%. Similarly, the shrinkage of PI-CP2 decreased compared to PI-C2. Because of the Si-O bond in the POSS backbone structure, a high bond energy makes it inherently stable and rigid [31]. As a result, the stability of the whole aerogel network was improved by adding POSS. Moreover, the porosity of composite aerogels was improved after the addition of CNTs and POSS, due to the inhibition of shrinkage and the nanopores provided by the cage structure of POSS [32]. The morphology of the aerogels was characterized, as shown in Figure 3b–f. As can be seen in Figure 3b, the pore size of the neat PI experienced a significant collapse due to the large shrinkage of the structure. The dimensions of the pores in the aerogels increased after they were made into composites with CNTs and CP (Figure 3c–f). This can be attributed to the presence of CNT and POSS, suppressing the shrinkage of the aerogels. Significantly, PI-CP2 exhibited the lowest shrinkage and the highest porosity, having the potential to be a thermal insulation material with excellent mechanical properties.

### 3.2. Mechanical Performance of PI Composite Aerogels

**Figure 4:**
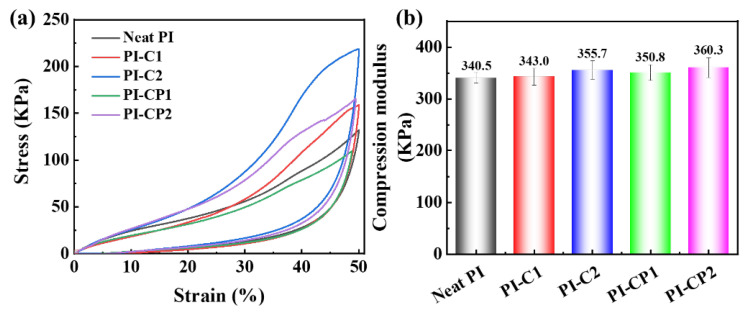
(**a**) Stress–strain curves and (**b**) compression modulus of PI aerogels.

Figure 4 shows the compressive properties of PI aerogels. Compared to neat PI, the compressive strength and modulus of PI-C1 increased from 128.4 and 340.5 KPa to 158.9 and 343.0 KPa, respectively. The compressive strength and modulus of PI-C2 further increased to 218.6 and 355.7 KPa, respectively. On the one hand, the high modulus of CNTs led to the improved mechanical properties of the aerogels [33]. On the other hand, the functional groups on CNTs effectively formed chemical and hydrogen bonds with the PI molecular chains, generating interconnected networks [34]. The superior compression performance of PI-C2 could be attributed to it having more functional groups, which increased the cross-linking degree of the aerogel. The effect of POSS on the compressive properties of the composite aerogels was also investigated. Compared to PI-C1, the compressive modulus of PI-CP1 increased from 343.0 to 350.8 KPa, while the compressive strength decreased from 158.9 to 109.4 KPa. A similar trend was also observed for PI-CP2 compared with PI-C2. The increase in modulus could be attributed to the incorporation of high-modulus inorganic POSS nanoparticles [35]. Furthermore, the grafting of POSS enhanced the surface roughness of the CNT, which improved the interfacial bonding with the PI matrix through physical interactions, leading to significant enhancement in the compressive modulus [36]. The decrease in compressive strength might be due to the increased porosity, making the aerogel more susceptible to structural damage under external forces.

### 3.3. Thermal Conductivity and Insulation Mechanism of PI Aerogels

**Figure 5:**
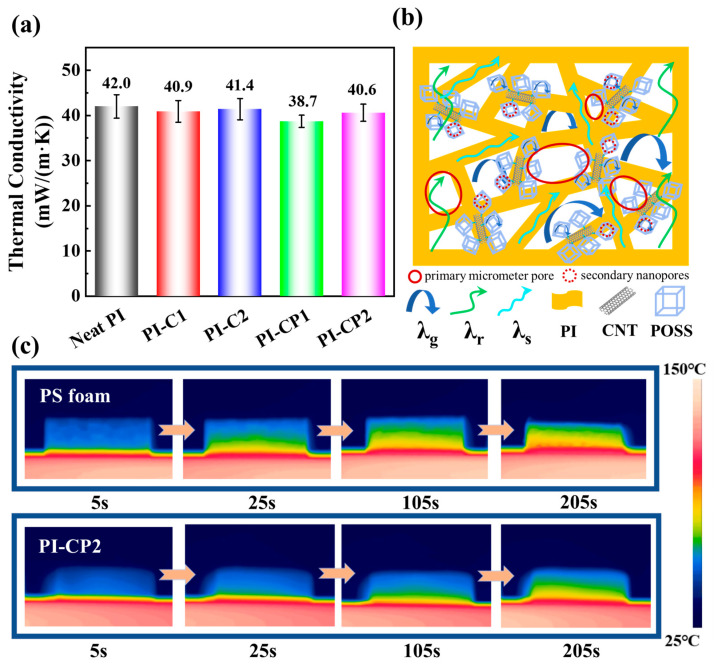
(**a**) The thermal conductivity of PI composite aerogels; (**b**) a diagram depicting the heat transfer processes within PI composite aerogels; (**c**) infrared images comparing the thermal profiles of PS foam and PI-CP2.

Figure 5a shows the thermal conductivity of the aerogels. Incorporating CNTs resulted in the thermal conductivity of PI-C1 decreasing to 40.9 mW/(m·K) compared to neat PI aerogel (42.0 mW/(m·K)). However, the thermal conductivity of PI-C2 increased to 41.4 mW/(m·K). According to the above analysis, the addition of CNTs increased the porosity of the aerogels, thus increasing the gas–solid interface of the aerogel, which in turn improved interfacial thermal resistance [37]. However, the superior thermal conductivity of the CNTs enhanced the solid-phase heat transfer [38], ultimately resulting in a higher thermal conductivity for PI-C2 than PI-C1. Furthermore, compared to PI-C1, the thermal conductivity of PI-CP1 was further reduced to 38.7 mW/(m·K). This change proved that POSS played a crucial role in improving the thermal insulation performance of the aerogels. Firstly, the hollow nanocage structure carried by POSS provided more nanoscale pores. Secondly, POSS improved the structural stability and inhibited the shrinkage of the aerogel, further increasing porosity. The above factors led to the enhanced gaseous conduction and weakened solid-phase conduction of the aerogel. Consequently, the PI-CP1 composite aerogels achieved outstanding heat insulation characteristics. The thermal conductivities of PI-CP2 and PI-C2 showed the same trend. However, the thermal conductivity of PI-CP2 exceeded that of PI-CP1, further suggesting that the high content of CNTs was not favorable to thermal insulation.

Figure 5b depicts the thermal transfer mechanism of PI-CP composite aerogels. The λ_g_, λ_r_ and λ_s_ in the figure refer to gaseous conduction, radiation conduction and solid-phase conduction, respectively. The structure of the composite aerogels consisted of primary micrometer pores (solid red circles) produced by freeze-drying and secondary nanopores (dashed red circles) provided by the cage structure of POSS. This multiscale pore structure provided an increased specific surface area, resulting in enhanced interfacial thermal resistance [39]. In addition, solid-phase conduction was attenuated due to the tortuous three-dimensional aerogel skeleton that extended the phonon transmission path. Furthermore, the contribution of radiation conduction was reduced by the introduction of CNTs due to the strong radiation absorption of CNTs [40,41].

Next, we compared the thermal insulation performance in practical applications of PI-CP2 with commercial polystyrene (PS) foam, as shown in Figure 5c. The temperature on the top side of the PS foam rose from 28.3 to 62.3 °C when it was placed on a 150 °C heated surface for a duration of 205 s. It can be seen that the PS foam structure was damaged, experiencing severe shrinkage. In contrast, the temperature at the top layer of PI-CP2 increased to 43.7 °C. Excellent structural performance and stability were observed, indicating that the thermal insulation capability of PI-CP2 significantly outperforms that of conventional PS foams.

## 4. Conclusions

In summary, CNTs grafted with POSS (CP) were used as reinforcement for the preparation of thermal insulating PI aerogels. The resulting aerogels with high porosity and low shrinkage were obtained by adding different amounts of CP. Benefiting from the physical and chemical cross-linking structures, excellent compression properties were achieved, resulting in PI-CP2 exhibiting a compressive strength of 167.7 KPa and a modulus of 360.3 KPa. Additionally, the unique multiscale pore structures increased the heat transfer path and reduced the thermal conduction at the interfaces, which in turn enhanced the thermal insulation properties of the aerogels, leading to PI-CP2’s low thermal conductivity of 40.6 mW/(m·K). The PI composite aerogels, with their exceptional thermal insulation and impressive compressive strength, hold great potential for advanced applications.

## Figures and Tables

**Table 1:** Characterization of the PI composite aerogels.

Samples	Density (mg/cm^3^)	Volume Shrinkage (%)	Porosity (%)
Neat PI	89.0	47.5	94.2
PI-C1	85.0	45.6	95.1
PI-C2	83.0	43.8	95.8
PI-CP1	78.0	41.7	96.7
PI-CP2	74.0	38.6	97.3

## Data Availability

Data are available upon request to the corresponding author.
